# The 3D-Printed (FDM/FFF) Biocomposites Based on Polylactide and Carbonate Lake Sediments—Towards a Circular Economy

**DOI:** 10.3390/polym15132817

**Published:** 2023-06-26

**Authors:** Robert E. Przekop, Ewa Gabriel, Marta Dobrosielska, Agnieszka Martyła, Paulina Jakubowska, Julia Głowacka, Piotr Marciniak, Daria Pakuła, Marek Jałbrzykowski, Grzegorz Borkowski

**Affiliations:** 1 Centre for Advanced Technologies, Adam Mickiewicz University in Poznań, 10 Uniwersytetu Poznańskiego, 61-614 Poznań, Poland; 2 Faculty of Materials Science and Engineering Warsaw, University of Technology, 141 Wołoska, 02-507 Warsaw, Poland; 3 Faculty of Chemical Technology, Institute of Technology and Chemical Engineering, Poznan University of Technology, 4 Berdychowo, 60-965 Poznań, Poland; paulina.jakubowska@put.poznan.pl; 4 Faculty of Chemistry, Adam Mickiewicz University in Poznań, 8 Uniwersytetu Poznańskiego, 61-614 Poznań, Poland; 5 Faculty of Mechanical Engineering, Bialystok University of Technology, 45c Wiejska, 15-351 Bialystok, Poland; 6 Faculty of Geographical and Geological Sciences, Adam Mickiewicz University, 10 B. Krygowskiego, 61-680 Poznań, Poland

**Keywords:** carbonate lake sediments, 3D printing, composite, FDM, injection moulding, PLA

## Abstract

In this study, composites containing polylactide and carbonate lake sediment in concentrations of 2.5, 5, 10, and 15% by weight were prepared by a 3D printing method. The material for 3D printing was obtained by directly diluting the masterbatch on an injection moulder to the desired concentrations, and after granulation, it was extruded into a filament. The material prepared thusly was used to print standardised samples for mechanical testing. To compare the mechanical properties of the composites obtained by 3D printing and injection moulding, two sets of tests were performed, i.e., mechanical tests (tensile strength, flexural strength, and impact strength) and hydrophobic–hydrophilic surface character testing. The degree of composite waste in the 3D printing was also calculated. Mechanical and surface tests were performed for both systems conditioned at room temperature and after accelerated ageing in a weathering chamber. The study showed differences in the properties of composites obtained by 3D printing. Sedimentary fillers improved the hydrophobicity of the systems compared with pure PLA, but it was not a linear relationship. The PLA/CLS sedB composite had higher strength parameters, especially after ageing in a weathering chamber. This is due to its composition, in which, in addition to calcite and silica, there are also aluminosilicates, causing a strengthening of the PLA matrix.

## 1. Introduction

Polylactide (PLA) is a linear polyester of lactic acid produced from substrates of natural origin, such as corn or sugar beets, and is characterised by its non-toxicity and biodegradability, making it safe for the environment. Polylactide has a low glass transition temperature, so it does not require high temperatures for processing, making it easy to use in both simple and more advanced printing equipment [1].

Polylactide is already widely used in a wide range of industries, from simple everyday applications, such as cups and cutlery, to more sophisticated applications, such as surgical threads and medical implants [2,3]. Polylactide can be modified relatively easily with various types of fillers to control its selected physicochemical, strength, or aesthetic properties [4]. From an environmental point of view, it is most expedient to modify this biopolymer with natural fillers, i.e., diatomaceous earth [5,6], cellulose [7], or wood [8]. In addition to these, the most common fillers used to improve the properties of polymeric materials include titania [9], talc [10], basalt fibers [11], and graphite [12].

Carbonates derived from natural lake and river sediments may also be an interesting filler for use in environmentally friendly polymer composites. To date, there have been several papers describing the effect of CaCO_3_ on the properties of PLA [13,14,15,16], especially in biomedical applications; however, the use of lake sediments for green composites, the effect of which is described in this paper, is a novel approach. The use of natural sediment in plastic processing is part of the circular economy. The circular economy is an economic model that aims to make rational use of resources and reduce the negative environmental impact of manufactured products. It represents an alternative concept to the currently dominant linear economy model [17]. The main three pillars of a closed-loop economy (CLE) are: reuse, reduce, and recycle. The closed-loop economy involves reducing the consumption of raw materials and reducing the waste generated, resulting in decreased consumption of the energy needed to produce new materials from scratch [18]. The CLE is a tool for sustainable development and can also contribute to economic benefits [19].

Objects obtained by additive FDM technology differ dramatically in terms of their structure from products produced by traditional processing techniques (injection moulding or compression moulding) due to, among other things, the presence of porous defects formed as a result of the imprecise overlapping of molten polymer layers, as well as high anisotropy of the properties resulting from the preference of a given direction of polymer paths in 3D printing. The structural factor resulting from the way objects are shaped causes significant differences in the mechanical properties of printed and injection-moulded products of the same geometry. The properties of printed objects are a result of the characteristics of the material and the print structure, which is not observed as much in other moulding techniques of plastic products [20,21,22,23].

**Figure 1:**
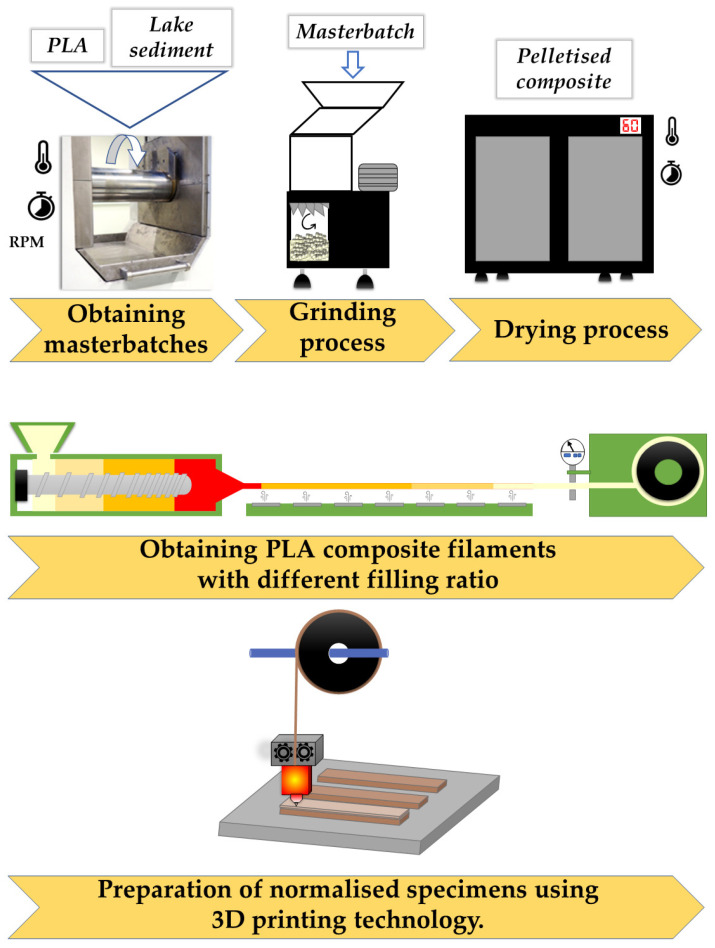
Diagram of the technological process of obtaining composites and forming objects by 3D printing (FDM).

This paper presents the effect of natural fillers (lake sediments) on the physicochemical properties of the PLA matrix composite materials obtained by 3D printing. In order to characterize the composites, a series of mechanical tests (static tensile strength, flexural strength, and Charpy impact strength) and microscope analyses (digital optical microscope and SEM-EDS) were carried out. In addition, contact angle measurements were performed to determine the hydrophilic/hydrophobic properties, and the waste coefficient was determined, which is important from the circular economy point of view. The tests were conducted for systems conditioned at room temperature, as well as in a weathering chamber. All the analyses were performed the same way as in our previous work, where the composites were obtained by injection moulding [24]. This approach allowed us to compare the impact of the two processing methods on the properties of the final product. The process diagram for obtaining the composites is shown in Figure 1.

## 2. Materials and Methods

### 2.1. Materials

Polylactide was used as a matrix polymer (PLA Ingeo™ Biopolymer 2003D, NatureWorks). The carbonate lake sediments (CLS) were collected from Lake Swarzędzkie in September 2017 at the geographical coordinates 52°24′50.5″ N; 17°04′14.8″ E.

### 2.2. Sample Preparation

#### 2.2.1. Preparation of Sediments

Fillers in the form of carbonate lake sediments were prepared in the same way as in the previous work [24]. Two fractions of sediments from a depth of 3–8 m (sediment A) and from a depth of 8–12 m (sediment B) were selected for comparison. The combined sediments were ground in the ball mill for 72 h and then fractionated using a vibrating sifter with sieves of different mesh sizes. For further testing, sediments with a particle size below 40 μm were used. Final concentrations of the filler in the tested systems are presented in Table 1.

#### 2.2.2. Preparation of Masterbatches

The process of homogenising polylactide (PLA) with fractionated sediments was carried out with the laboratory two-roll mill ZAMAK MERCATOR WG 150/280. The amount of 1000 g of PLA was plasticised at 215 °C for 15 min while portions of sediments were added until a 50% *w*/*w* filler concentration by weight was reached. Then, the batches were ground in the WANNER C17.26 SV mill and dried for 24 h at 60 °C. The 50% PLA masterbatch was diluted with peat polylactide in appropriate weight ratios and injected using an Engel e-victory 170/80 injection moulding machine to obtain the following filler concentrations: 2.5%, 5%, 10%, and 15%. The injection moulding process was carried out in the temperature range of 185–200 °C. The resulting test specimens were ground in a plastic mill and dried for 24 h at 60 °C. The resulting granules were used to make 3D printing filament with a FILABOT EX60 micro extruder. The temperature of extruder zones in extrusion process are shown in Table 2. They were selected individually for each material, depending on the filler content.

#### 2.2.3. Preparation of Samples

Standard test specimens were printed using a Creality Ender 3 3D printer. The printing parameters are summarised in Table 3. Models of the standard test objects were prepared for printing with the Creality Slicer 1.2.3 program. On the basis of the implemented models, the G-code files for the 3D printer were generated.

### 2.3. Characterisation Methods

Water contact angle (WCA) measurements were performed using the sessile drop technique (5 µL) at room temperature and atmospheric pressure with a Krüss DSA100 goniometer (Krüss Optronic GmbH, Hamburg, Germany).

Differential scanning calorimetry (DSC) was performed using a NETZSCH 204 F1Phoenix (NETZSCH, Selb, Germany). Samples of 4 ± 0.2 mg were placed in an aluminium crucible with a punctured lid. The measurements were performed under nitrogen in the temperature range of 20–220 °C and at a 10 °C/min heating rate. The measurements were carried out in two cycles. The T_g_ temperature was determined from the second cycle.

The ageing tests (CCh) were performed in an ESPEC ARS-0220 (ESPEC, Pyeongtaek, Republic of Korea) weathering chamber with ten −10 °C to +50 °C alternate cooling and heating cycles (five days total) and at a humidity of 85%.

For flexural and tensile strength tests (in accordance with PN-EN ISO 178 and PN-EN ISO 527 standard, respectively), the produced materials were printed into type 1B and 1BA specimens. The tests were performed on a universal testing machine, Instron 5969. The traverse speed for both types of measurements was 2 mm/min.

The Charpy impact strength test (unnotched samples) was performed according to ISO 179-1 on an Instron Ceast 9050 impact pendulum tester.

For the data obtained from the strength tests, an ANOVA analysis (Anova in R—Stats and R) was performed—the multivariate analysis of variance with the aov function from the stats package.

Images of composite surface and fractures were taken with the digital light microscope Keyence VHX-7000 (Keyence International NV/SA, Osaka, Japan) with a VH-Z100R wide angle zoom lens (Keyence International NV/SA, Osaka, Japan) at 100× and 300× magnification. The images were taken using the function of depth composition and 3D image creation. Standard coaxial lighting was used.

SEM/EDS analyses were recorded on a Quanta FEG 250 (FEI) instrument operating at 5 kV (SEM) and at 30 kV (EDS) voltage.

The phase identification and the relationship between the sediment fraction depth and its composition were determined using an X-ray diffraction (XRD) powder diffractometer Smartlab SE Rigaku (Rigaku, Tokyo, Japan) using CuKα lamp radiation and a Ni filter. X-ray diffractograms were recorded in the angular range of 3–100° (2θ).

Differential scanning calorimetry (DSC) was performed using a NETZSCH 204 F1Phoenix (NETZSCH, Selb, Germany). Samples of 4 ± 0.2 mg were placed in an aluminium crucible with a punctured lid. The measurements were performed under nitrogen in the temperature range of 20–220 °C and at a 10 °C/min heating rate. The measurements were carried out in two cycles. The T_g_ temperature was determined from the second cycle.

## 3. Results and Discussion

### 3.1. Mechanical Tests

**Figure 2:**
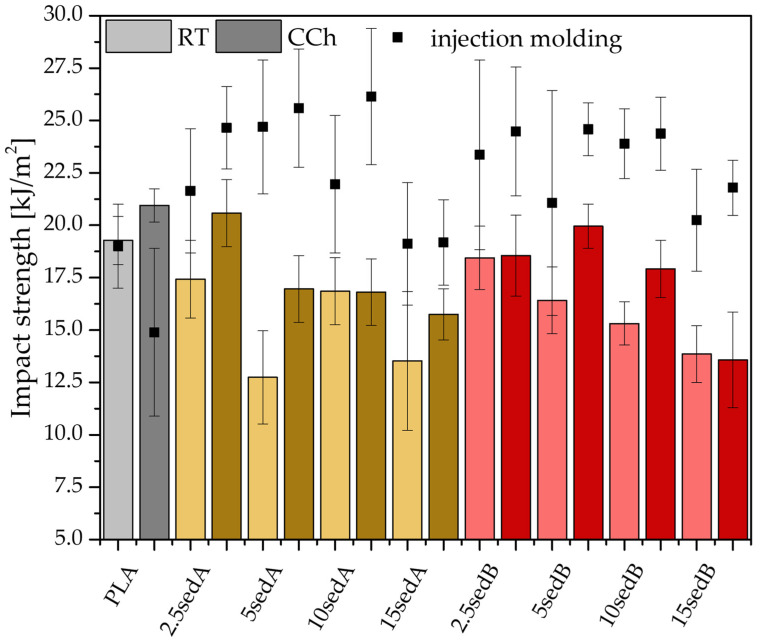
Impact strength of composites obtained by 3D printing; RT—room temperature, CCh—climate chamber.

**Figure 3:**
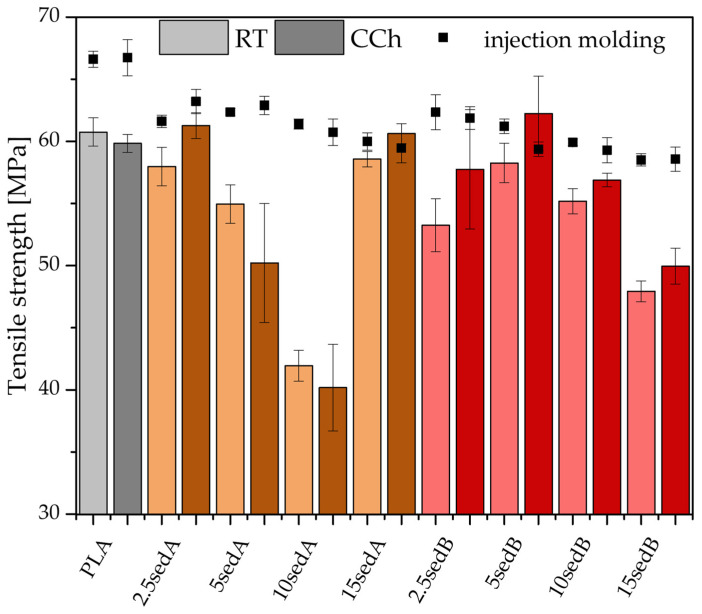
Tensile strength of printed composites; RT—room temperature, CCh—weathering chamber.

**Figure 4:**
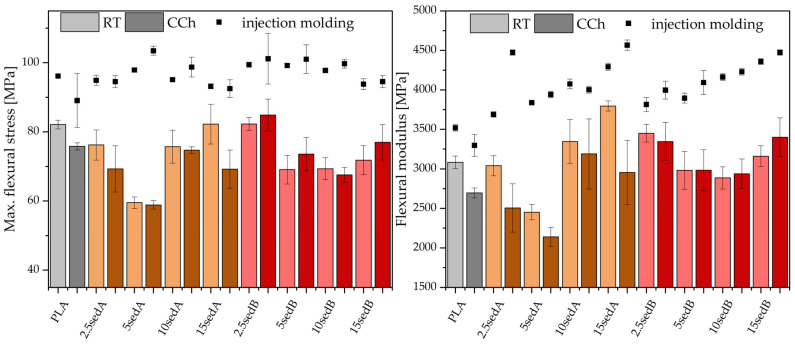
Maximum flexural stress of printed composites (**left**); Flexural modulus of printed composites (**right**); RT—room temperature, CCh—weathering chamber.

The PLA/CLS composite samples printed using the FDM technique were tested for impact, tensile, and flexural strength properties, both after conditioning at room temperature and after accelerated ageing in a weathering chamber—Figure 2, Figure 3 and Figure 4.

In the previous paper, similar tests were carried out on samples produced by injection moulding [24]. Impact strength of neat-injection-moulded polylactide samples was approximately 19 kJ/m^2^, while after accelerated ageing in the weathering chamber, it decreased by 5 kJ/m^2^. The impact strength of the reference 3D-printed sample was 19.3 kJ/m^2^ and approximately 21 kJ/m^2^ after ageing in the weathering chamber (Figure 2). As in the neat PLA samples, most filled samples of both types showed some reinforcement after accelerated ageing in the weathering chamber. CLS filler introduced a gradual decrease in impact strength with its content; however, sedA CaCO_3_-rich systems generally showed worse properties than sedB systems with mixed components filler (additional hardening effect of silica and aluminosilicates).

Cyclic conditioning at temperatures reaching +50 °C, close to the T_g_ of PLA, may have caused an increase in the ratio of the PLA crystalline phase with better mechanical properties than the amorphous one. This was also observed for most of the other mineral-filler-containing PLA systems tested and for the other printed objects subjected to varying temperatures [25,26]. The lower impact strength of filled samples than that of neat PLA can be explained by the presence of internal notches in the material resulting from weak interactions at the interfacial boundary between the mineral filler grains and the polymer matrix. Mineral fillers, e.g., aluminosilicates, often agglomerate in the polymer matrix [27]. Impact strength tests have shown that composites containing SedA and SedB have similar impact strengths in general. However, the broad results distribution introduced by the high anisotropy of the properties in such systems hinders a proper assessment of the filler effect [28].

The tensile strength of a neat printed polylactide exhibited a value of 60 MPa, both before and after the accelerated weathering test (Figure 3). This is slightly lower than for the injected samples. Significant differences were observed for the sediment-filled systems, especially in the sedA series.

The addition of the CLS fillers used did not significantly impair the tensile strength of the printed samples. A decrease in strength below 55 MPa occurred only at high filler content in the polymer matrix (10% SedA and 15% SedB). For all systems tested, a slight improvement in strength was observed after CCh, with the exception of the 10sedA composition in the PLA samples (Figure 3).

The elasticity of PLA/CLS composites on the base of elongation at break is lower compared with the unmodified polymer. This decrease in elasticity is proportional to the increase in stiffness (Young’s modulus) caused by the addition of the sediments (Table 4). After the accelerated ageing in the weathering chamber, the stiffness of the SedB specimens remained at the same level, while an increase in elasticity was observed. Composites containing SedA show deteriorated stiffness as a result of being in the weathering chamber. The tensile behavior of the pure polymer does not change significantly as a result of ageing in the weathering chamber.

On the basis of flexural strength tests, it can be concluded that the presence of sedB results in higher elasticity compared with the composite samples with sedA. Moreover, the parameters obtained for the composite with sedB are higher compared with the tests conducted for neat PLA (Figure 4).

The flexural strength and stiffness of 3D-printed neat PLA deteriorated due to the cyclic exposure to varying temperatures. The 15sedA- and 2.5SedB-printed composites exhibited flexural strength at the level of the neat PLA. Conditioning the SedA samples in a weathering chamber resulted in a decrease in flexural strength relative to the reference materials conditioned at room temperature (RT). The stiffness of PLA after the introduction of fillers to the polymer matrix remained unchanged or improved for most of the tested printed samples (except for 5SedA). The flexural modulus decreased for PLA and samples containing SedA after CCh. Young’s (flexural) modulus of the SedB composites was kept relatively constant throughout the filler concentration.

The phenomenon of improvement of the mechanical properties after treatment in the climate chamber was also observed for the samples that were obtained by the injection moulding technique [24]. The operation of the climate chamber can be regarded as a short-term conditioning of the samples, which had a positive effect on the mechanical properties of the PLA/CLS composites.

**Figure 5:**
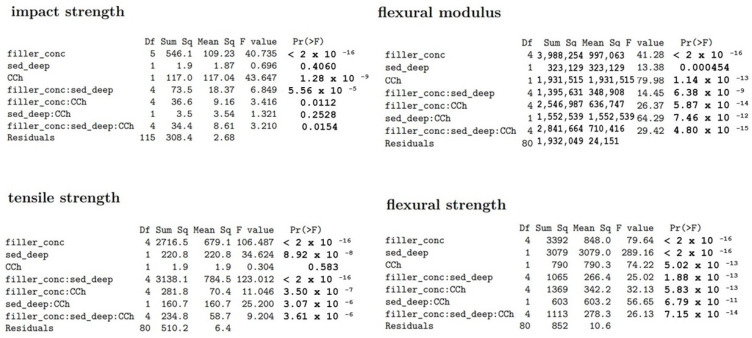
The results of ANOVA analysis.

The ANOVA (ANalysis Of VAriance) is a statistical test to determine whether two or more population means are different. In other words, it is used to compare two or more groups to see whether they are significantly different. The results of the stress tests were grouped into sets according to lake sediment content, sediment sampling depth, and the effect of climate chamber operation, and a multivariate analysis of variance with the aov function from the stats package was performed. The results obtained in the strength tests were analysed, and the results are shown in Figure 5.

The results of the analysis of variance indicated the rejection of the hypothesis of equality of means and allowed us to assume that all values differed in a statistically significant way. The highest differences were shown in the CCh score group for flexural modulus (F = 79.98), sampling depth for flexural strength (F = 289.16), filler concentration: sampling depth for tensile strength (F = 123.012), and CCh score group for impact strength (F = 43.647).

### 3.2. Waste Factor

**Figure 6:**
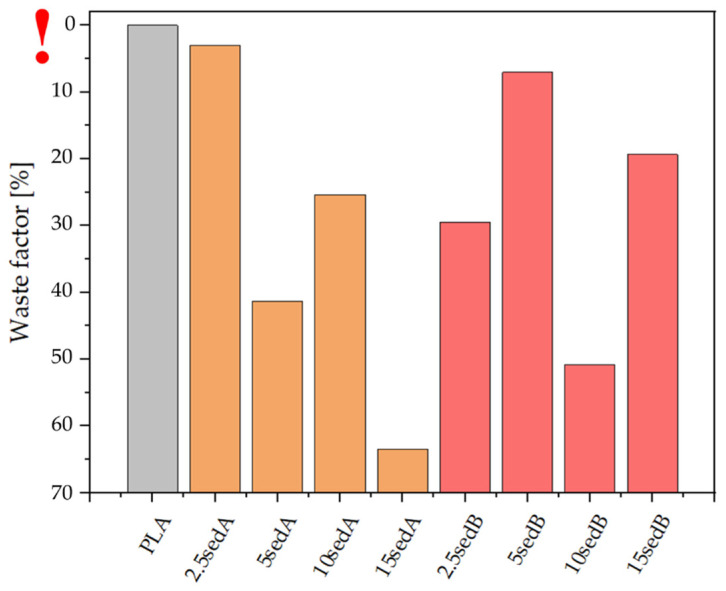
Waste factor (the exclamation mark indicates the position of 0 on the Y axis).

The waste factor for neat polylactide is nearly 0% (Figure 6), which means that no loss of the material was observed during the fabrication of the samples by FDM printing. Printing difficulties were observed for the systems containing 5% and 15% of sedA and 10% of sedB. The remaining samples had a waste factor of no less than 30%. For the sedB modifier, the high filling ratio, i.e., 15%, did not cause major difficulties in 3D printing, as the waste factor was approximately 20%. The uneven diameter of the filament and its brittleness significantly affected the 3D printing process; hence, the higher the filler content, the higher the material loss. The waste coefficient correlated with the strength properties in mechanical tests and could explain the abrupt deterioration of print parameters, such as the steep decrease in flexural strength for 5SedA or the lack of a constant trend with respect to the change of filler content in the PLA matrix.

### 3.3. Surface Properties—Contact Angle Measurements

The hydrophobic–hydrophilic properties of the composite surface were determined both for the samples conditioned at room temperature and those aged in a weathering chamber. Table 5 shows the contact angle values for the PLA SedA and SedB composites. All the composites tested had hydrophilic surfaces (contact angle < 90°), regardless of the type and concentration of the filler, which is due to the properties of PLA and the filler themselves. There is no clear correlation between the composition and contact angle in the two series. Another factor that caused significant differences in values is the structure and microstructure of the surfaces obtained by 3D printing. Defects in the structure of the samples are described in Section 3.4: Composite surface morphology—optical and electron microscopy. Ageing in the climate chamber caused an increase in the surface irregularities of the composite, which resulted in a decrease in the surface contact angle. In the case of the sedA filler, a greater reduction in the contact angle value was observed, which was due to the presence of a higher amount of calcium carbonate compared with the SedB filler samples [29], which were highly hydrophilic [30]. The decrease in contact angle was almost 20° in some cases.

WCA measurements were carried out to investigate the effect of the filler on the wettability of composites. In general, the hydrophobicity of the composites increased after the addition of a sedimentary filler relative to pure PLA, although this was not a linear relationship. There was also an increase in resistance to external conditions, as evidenced by the improvement in mechanical properties after the climatic chamber.

### 3.4. Composite Surface Morphology—Optical and Electron Microscopy and SEM/EDS Mapping

**Figure 7:**
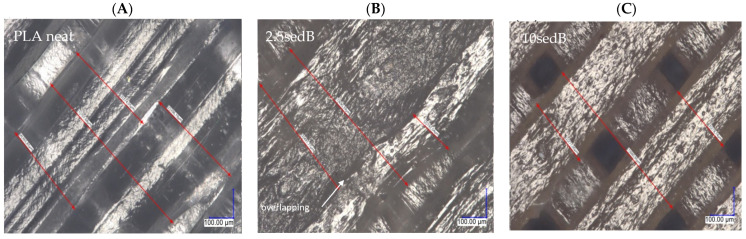
Images of the surface of printed bars (300× magnification), (**A**) neat PLA, (**B**) 2.5sedB, (**C**) 10sedB (red arrows indicate how the width of the print path is measured).

All images of the surface of the samples were taken at 300× magnification. A portion of the center part of the surface of the sample was analyzed, i.e., in the middle of the width and in the middle of the length, resulting in the observation of only the filling line of the top layer of the 3D model in the images. No changes were observed in the surface structure of the samples after ageing in the weathering chamber, either for the PLA reference sample or the composites with carbonate lake sediments. The filler agglomerates resulted in surface roughness of the printed composites, and their number and diameter increased with filler concentration. Figure 7 shows the layer thickness and distance measurements.

Table 6 summarizes the fill line width measurements for two adjacent lines for each sample.

In the 3D printing process, a nozzle with a diameter of 400 μm was used, so the fill line width of the printed samples should have been close to this value. Neither measurement of the fill line width for the neat PLA reference sample without filler additive differed much from the value set in the cutting program (“slicer”). For the composite samples with sediments, the fill line widths were significantly lower and deviated from the nominal nozzle diameter (400 μm).

The lowest, as well as the highest, values of fill line width were recorded for the 2.5sedB sample. This error was due to the partial overlap of the two lines. The significant differences in the fill line width values of the composite samples were due to changes in the rheological properties of the materials melted in the printer nozzle, as well as to the inhomogeneity of the composite and the lower dispersion of the filler in the polymer matrix. These factors were able to affect the uniformity of material dispensing during the printing process, resulting in uneven structures and microstructures.

**Figure 8:**
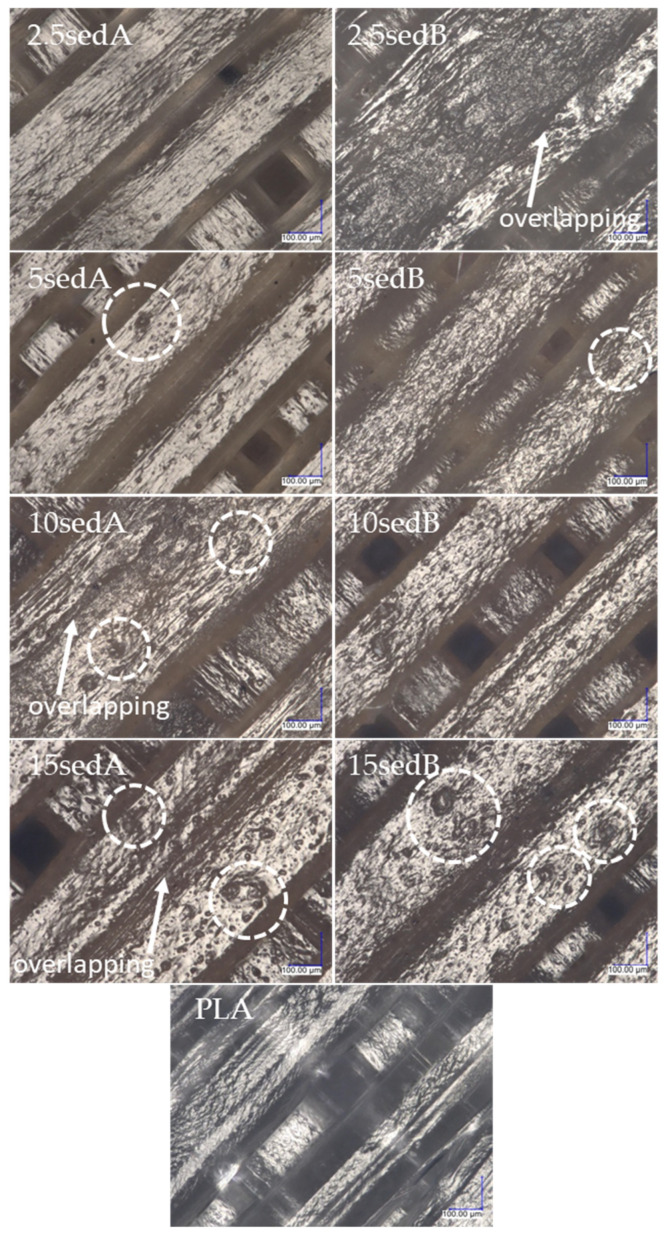
Images of surface structures of printed samples (highlighted areas indicate filler agglomerates).

**Figure 9:**
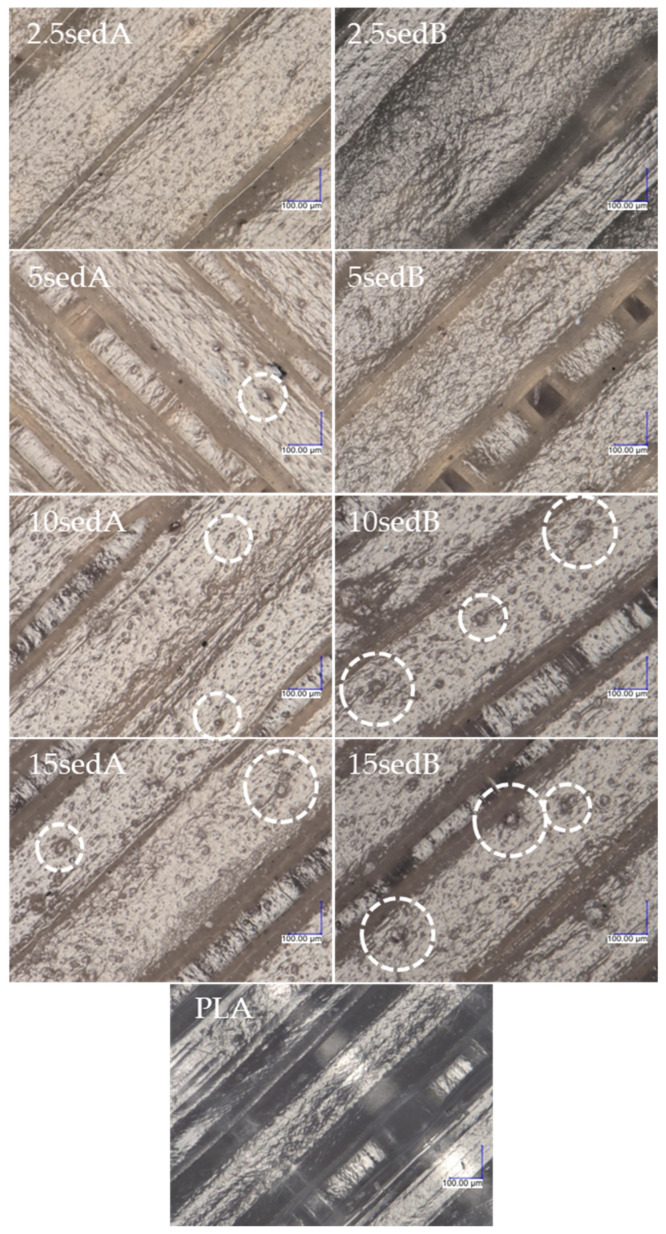
Surface structures of composites after testing in a climate chamber (highlighted areas indicate filler agglomerates).

Figure 8 and Figure 9 show images of the surfaces of FDM-printed samples taken with a digital optical microscope before (Figure 8) and after ageing in the weathering chamber (Figure 9).

**Figure 10:**
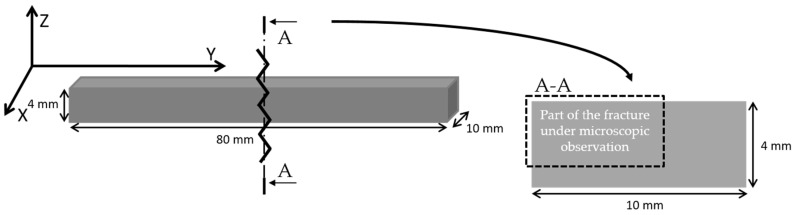
Diagram illustrating the placement of the shaper in the working field of the 3D FDM printer, the location of the fracture after the impact test, and the area of the cross section under observation.

Observations of the breakthroughs of the samples were also made. Microscopic analysis was performed at the upper left corner of the cross section (Figure 10), so the images taken show two outline lines on the left side of the photo and model fill lines arranged at a 45° angle to the outline. The images were taken using the 3D depth composition function and 100× magnification.

**Figure 11:**
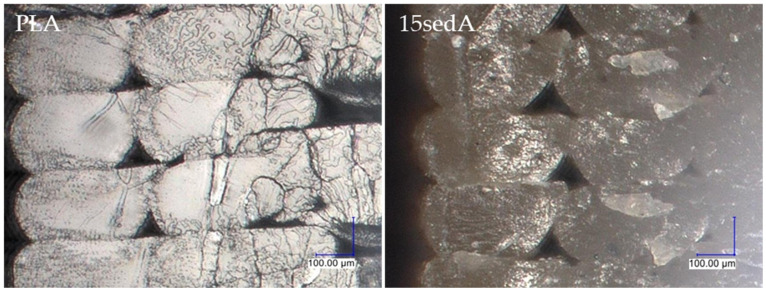
Enlarged photo of the breakthroughs of the contour lines of the PLA sample and the composite with 15% filler content (sedA).

**Figure 12:**
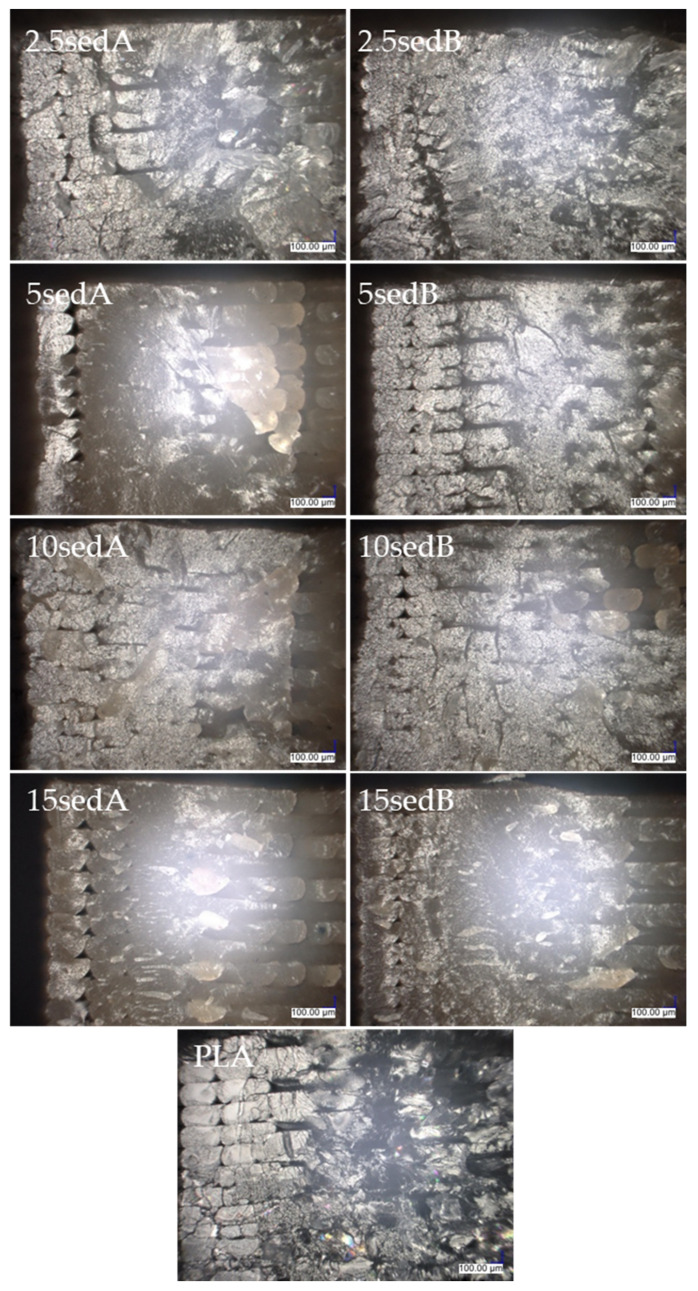
The structures of composite cross sections.

**Figure 13:**
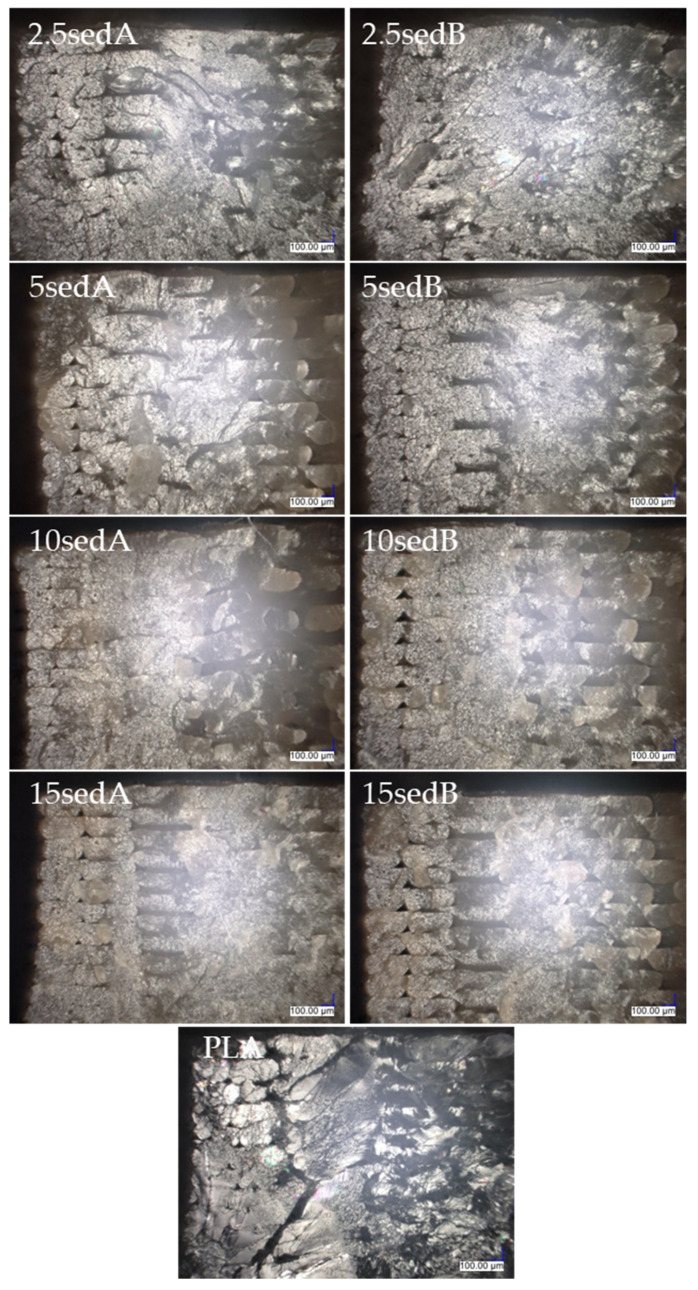
The structures of composite cross sections after conditioning in a climate chamber.

Figure 11 shows an image of the breakthrough cross section of the contour lines of a PLA sample and a composite with 15% filler content. Figure 12 and Figure 13 show images of the cross section of the specimens after the impact strength test prior to and after ageing in the weathering chamber. The contour lines break transversely to their length during the impact, so the cross-sectional shape of the successively superimposed contour lines, the size of the voids between them, and where they overlap can be seen. Comparing the photos of specimens before and after accelerated ageing in the climatic chamber, no significant differences are observed. Where the contour lines overlap with the filler lines, the breakthrough structure is noticeably more uniform, with no voids, compared with the breakthrough structure closer to the center of the sample. As the filler content increases, the fracture lines disappear.

**Figure 14:**
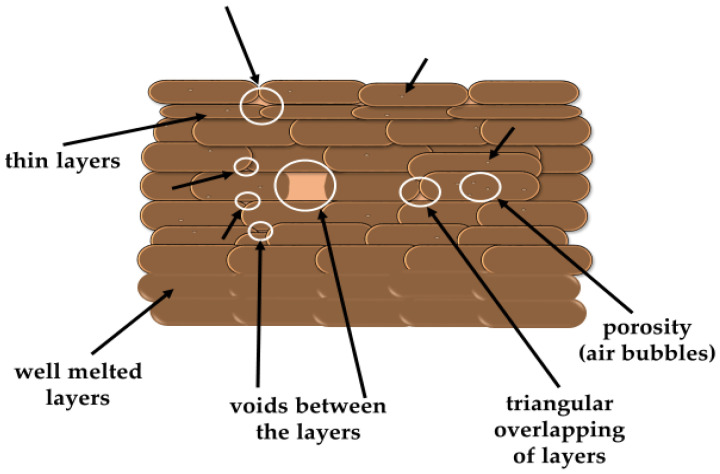
Types of defects observed in 3D-printed objects.

Under microscopic observations, the defects in the composites structure were seen, which may have been a result of both the processing and the filler used. Examples of the possible types of defects are shown schematically in Figure 14.

**Figure 15:**
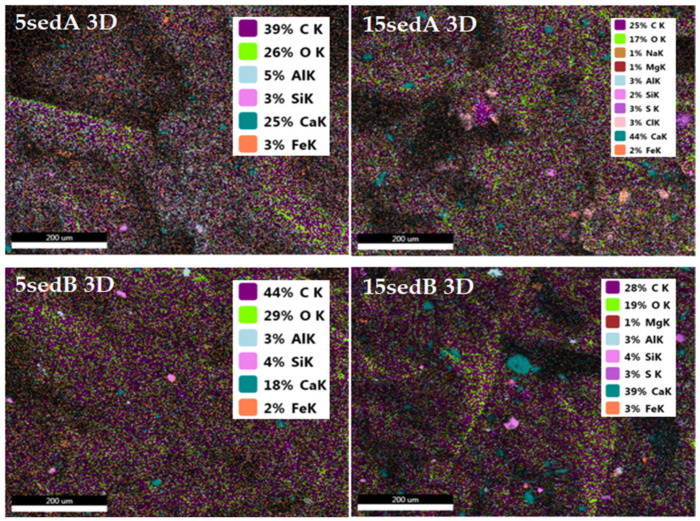
EDS mapping of 3D-printed samples.

EDS mapping of elements for fractures of the printed 3D composite samples allowed the determination of the distribution of filler particles in the PLA matrix (Figure 15). The dispersion of sediments particles in the matrix was not homogenic. Similar results were obtained in our previous work with injection moulding samples [24]. The highest structure heterogenicity (based on the distribution of calcium) was in the 15sedB system.

The main components of SedA and SedB were CaCO_3_ and SiO_2_ [24]. However, as the sampling depth increased, components such as mullite, kaolinite, muscovite, anhydrite, dolomite, or aluminosilicate, and sulfate (SedB) also appeared in the sample [29]. Diffractograms of 3D-printed composites with SedA and SedB showed reflections originating from PLA at angles of 14, 16, and 32 (2 θ), indicating the presence of the alpha crystalline form of PLA and a broadening corresponding to the amorphous form of PLA (heat treatment in FDM produces amorphous PLA as a non-crystalline peak [31]).

**Figure 16:**
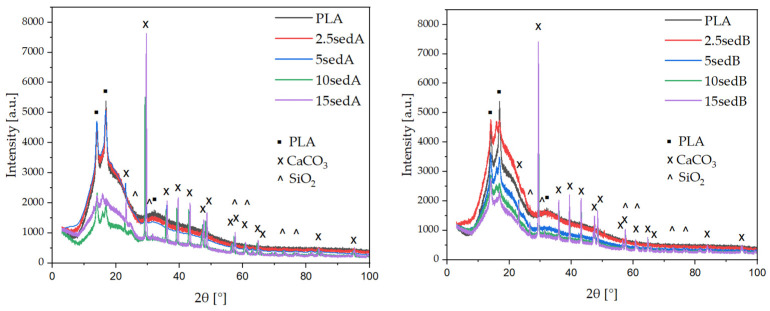
XRD diffractograms of 3D-printed samples.

PLA is characterised by slow crystallisation kinetics often observed in conventional processing methods (extrusion, injection moulding, etc.). This phenomenon has a significant impact on the thermal and mechanical properties of the final product. In many applications, it is desirable to increase the crystallinity of PLA, since in amorphous form, the use of PLA is limited by its low glass transition temperature (T_g_). At temperatures higher than T_g_, only the crystalline phase of PLA can impart valuable mechanical properties. Thus, the crystalline form is necessary to increase the strength of the material [32,33]. Typically, the PLA homopolymer has three crystalline forms [32,33,34], namely α-, β-, and γ-form, which depend on the crystallisation conditions. Among all these crystalline forms, the α-form is the most common and stable polymorph. Thanks to the presence of the α-structure, we can expect an improvement in the mechanical properties of the composites. Moreover, the remaining reflections originated from CaCO_3_, and their intensities increased as the filler content increased (Figure 16).

The presence of SiO_2_, aluminosilicates, and other minerals, as well as lower organic matter content in the precipitate derived from deeper parts, positively affected the mechanical properties of the composites with these fillers [35]. The tensile strength, or Young’s modulus, had higher values compared with the composites with SedA.

Differential scanning calorimetry analysis was carried out for the 3D-printed samples. Measurements were carried out in the temperature range of 20–220 °C and at a 10 °C/min heating rate in an inert gas flow. The characteristic temperatures of phase transition were determined from the second heating cycle. On the basis of the DSC analysis, the effect of fillers on the phase transitions in composites was determined.

**Figure 17:**
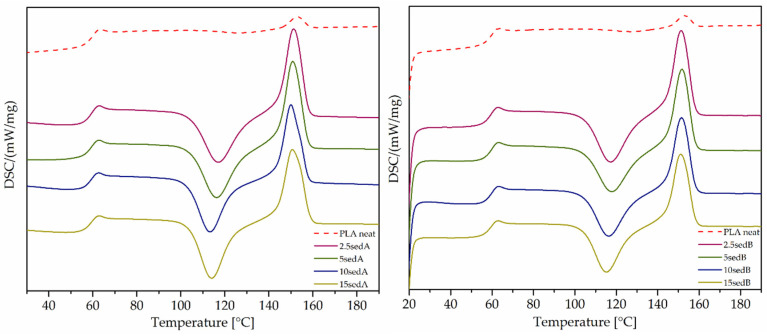
DSC thermograms of 3D-printed samples.

Three characteristic peaks can be observed in the presented thermograms (Figure 17), which originated from the glass transition temperature T_g_ (range 50–70 °C), the cold crystallisation temperature T_cc_ (range 100–130 °C), and the melting temperature T_m_ (range 140–165 °C). These phase transitions are characteristic of semicrystalline polymers, which include polylactide, which has both an amorphous and crystalline phase.

The addition of fillers did not significantly affect the T_g_ and T_m_ of the composites obtained. The biggest difference can be seen for the cold crystallisation (T_cc_). The thermograms clearly show a peak T_cc_ for neat polylactide that was hardly noticeable, while the addition of even the lowest precipitate concentration (2.5%) caused a significant change. Narrowed signals of cold crystallisation and lower temperatures were evident for the composites (PLA neat T_cc_ = 127.8 °C; 10sedA T_cc_ = 113.1 °C). The pronounced, sharp T_cc_ peaks of the composites compared with a reference sample of neat PLA indicated higher crystallinity for the materials with fillers. The DSC analysis indicated that the applied deposits in the polylactide matrix exhibited the nucleating properties. Analogous results were obtained in our previous work [24].

## 4. Conclusions

This paper presents the study results of the effect of natural lake sediments in PLA matrix composite materials and refers to the comparison of processing methods (injection moulding and 3D printing) and their impact on the parameters of the final materials. The fillers used, carbonate lake sediments, were of natural origin. The effect of the presence of fillers on the textural and mechanical properties of PLA was investigated. Tests showed that the PLA/CLS sedB composite had higher strength parameters, especially after ageing in a weathering chamber. SedB is a sediment taken from greater depths (8–12 m), and apart from calcite, it also contains aluminosilicates, causing a strengthening of the PLA matrix. Cyclic exposure of the samples with SedB in a weathering chamber to temperatures near the PLA glass transition temperature had a positive effect on some mechanical properties of the tested printed objects (impact and flexural strength). It can be said with certainty that natural fillers can find applications in the field of PLA composites. To improve the interfacial interactions of the PLA matrix and the mineral fillers (CLS), the authors plan to extend their research to introduce surface functionalisation of the fillers. The presented work is also a continuation and extension of the research on the use of natural sedimentary mineral filler in the conventional processing of plastics [36,37,38].

## Figures and Tables

**Table 1:** Final concentrations of the filler in the tested systems.

Sample Name	Concentration of Filler (%)
2.5sedA	2.5, 3–8 m sediment, <40 µm
5sedA	5, 3–8 m sediment, <40 µm
10sedA	10, 3–8 m sediment, <40 µm
15sedA	15, 3–8 m sediment, <40 µm
2.5sedB	2.5, 3–8 m sediment, <40 µm
5sedB	5, 3–8 m sediment, <40 µm
10sedB	10, 3–8 m sediment, <40 µm
15sedB	15, 3–8 m sediment, <40 µm

**Table 2:** Parameters of the filament extrusion process for 3D printing.

Sample Name	Temperature (Die/Front/Middle/Feed) (°C)
2.5sedA	190/190/195/55
5sedA	180/200/200/55
10sedA	180/185/195/55
15sedA	163/185/195/55
2.5sedB	163/185/195/55
5sedB	163/185/195/55
10sedB	170/195/190/60
15sedB	185/200/200/60

**Table 3:** Parameters of 3D printing of CLS/PLA composites.

Layer height	0.2 mm
Printing speed	60 mm/s
Printing speed of the 1st layer	20 mm/s
Printing temperature	210 °C
Bed temperature	60 °C
Nozzle diameter	0.4 mm
Sidewall thickness	0.8 mm
Infill percentage	100%

**Table 4:** Elongation at break and Young’s modulus of printed composites; RT—room temperature, CCh—weathering chamber.

Sample	Young’s Modulus (Mpa)		Elongation at Break (%)	
	RT *	CCh *	RT *	CCh *
Neat PLA	3062 ± 94	2897 ± 94	2.61 ± 0.13	2.82 ± 0.13
2.5sedA	3330 ± 67	2988 ± 65	2.31 ± 0.23	2.70 ± 0.22
5sedA	3220± 157	2704 ± 212	2.36 ± 0.22	2.52 ± 0.13
10sedA	2690 ± 52	2295 ± 240	2.10 ± 0.11	2.15 ± 0.25
15sedA	3916 ± 28	3661 ± 96	2.25 ± 0.09	2.55 ± 0.20
2.5sedB	3054 ± 94	2805 ± 289	2.34 ± 0.09	2.81 ± 0.14
5sedB	3456 ± 68	3218 ± 244	2.27 ± 0.11	2.53 ± 0.24
10sedB	3463 ± 85	3728 ± 131	2.34 ± 0.06	2.72 ± 0.23
15sedB	3378 ± 30	3222 ± 125	2.05 ± 0.11	2.44 ± 0.18

* RT—room temperature, CCh—after weathering chamber.

**Table 5:** Contact angle measurements.

Sample	Contact Angle (°)	
	RT *	CCh *
Neat PLA	79.2 ± 1.7	77.2 ± 3.4
2.5sedA	86.4 ± 2.0	70.0 ± 1.8
5sedA	77.4 ± 1.6	76.4 ± 3.8
10sedA	80.2 ± 2.6	67.4 ± 4.8
15sedA	86.1 ± 4.1	67.6 ± 5.0
2.5sedB	80.3 ± 1.5	64.3 ± 3.7
5sedB	85.9 ± 2.3	80.9 ± 2.3
10sedB	73.2 ± 4.5	72.6 ± 3.0
15sedB	84.0 ± 1.9	79.1 ± 1.5

* RT—room temperature, CCh—ageing in the weathering chamber.

**Table 6:** The width of the fill lines of the printed specimens not tested in the climatic chamber measured with a digital microscope; the values are given in unit (μm).

Sample Name	Fill Line 1	Fill Line 2
PLA	396	365
2.5sedA	342	301
5sedA	322	286
10sedA	395	208
15sedA	350	282
2.5sedB	497	192
5sedB	333	315
10sedB	277	278
15sedB	378	332

## Data Availability

Not applicable.
